# Pulsed Field Ablation for Ventricular Arrhythmia in Repaired Tetralogy of Fallot

**DOI:** 10.1016/j.jaccas.2025.106335

**Published:** 2025-12-11

**Authors:** Nili Schamroth Pravda, Lorenzo Caratti di Lanzacco, Rui Shi, Ghada D. Mustafa, Sabine Ernst

**Affiliations:** a Department of Cardiology, Tel Aviv Medical Center-Ichilov, Affiliated to Gray Faculty of Health Sciences, Tel Aviv University, Tel Aviv, Israel; b Department of Cardiology, Royal Brompton Hospital, London, United Kingdom

**Keywords:** ablation, tetralogy of Fallot, ventricular tachycardia

## Abstract

**Background:**

Ventricular arrhythmias are a major late complication in patients with repaired Tetralogy of Fallot (TOF), frequently originating from slow conduction zones within surgical isthmuses. Pulmonary valve replacement (PVR) may limit future ablation access, prompting interest in pre-emptive substrate modification. Pulsed field ablation (PFA) is a novel nonthermal ablation modality with potential safety advantages.

**Case Summary:**

A 45-year-old man with repaired TOF and severe pulmonary regurgitation underwent a pre-PVR electrophysiology study, which identified a slow conduction zone at isthmus 3, located between the right ventricular outflow tract and the ventricular septal defect patch. Focal PFA achieved bidirectional block without complications.

**Discussion:**

This first reported use of focal PFA for ventricular arrhythmia substrate modification in TOF demonstrates its feasibility and short term safety, supporting its potential role in pre-emptive arrhythmia management before structural interventions.

**Take-Home Messages:**

This case demonstrates the feasibility of using focal PFA for the treatment of ventricular arrhythmia in patients with repaired TOF. It highlights the role of pre-emptive substrate modification before PVR in patients with repaired TOF.

## Graphical Abstract

**Figure:**
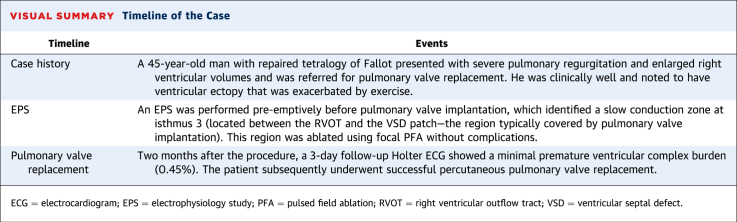

Patient with tetralogy of Fallot (TOF) are at increased risk of sudden cardiac death due to ventricular arrhythmias.^1^ Recent studies have advanced the understanding of the pathophysiology of ventricular ectopy and ventricular tachycardia (VT) in TOF, particularly emphasizing the role of anatomical re-entry circuits known as *electrical isthmuses*.^1^ These isthmuses are regions of slow conduction and can be targeted by arrythmia ablation.

Four distinct isthmuses have been described in TOF. Among these, isthmus 3, located between the right ventricular outflow tract (RVOT) and the ventricular septal defect (VSD) patch, is most frequently implicated.

Its narrow width, thin myocardial wall, and frequent involvement of surgical scar make it highly susceptible to slow conduction and re-entry, explaining its predominant role in VT circuits.^2^

Importantly, isthmus 3 often becomes inaccessible after pulmonary valve replacement (PVR), further highlighting the rationale for pre-emptive substrate modification in this setting.^3^

Pulsed field ablation (PFA) is an emerging ablation technology that delivers high-energy pulsed electric fields to induce irreversible electroporation, achieving tissue-selective myocardial ablation without the thermal injury associated with conventional radiofrequency (RF) or cryothermal energy.^4^ Although PFA has shown early promise in the treatment of ventricular arrhythmias,^5^ we report the first use of focal PFA for the treatment of ventricular ectopy in a patient with repaired TOF.

## History of Case Presentation and Past Medical History

A 45-year-old man with TOF, after surgical repair in childhood with a monocusp reconstruction of the RVOT, presented with severe pulmonary regurgitation and enlarged right ventricular volumes.

## Investigations

Cardiac magnetic resonance imaging demonstrated an indexed right ventricular diastolic volume of 152 mL/m^2^, with moderate right ventricular hypertrophy and preserved right ventricular function, with an ejection fraction of 58%. Also noted was a 38-mm region of akinesia below the RVOT, concurred with the region of the transannular patch.

The patient underwent a cardiopulmonary exercise test. He was able to exercise for 12 minutes 54 seconds, achieving a peak oxygen consumption of 24.7 mL/kg/min (66% of predicted), with ventricular ectopy including couplets on exercise with a 7-beat run of nonsustained VT during Bruce stage 5 and a maximum 5-beat nonsustained VT in early recovery. The main morphology was left bundle branch block–like, with transition at lead V_3_/V_4_ and an inferior axis (see baseline electrocardiogram [ECG] in Supplemental Figure 1 and ECG during exercise in Supplemental Figure 2). He was treated with a β-blocker (bisoprolol 2.5 mg once daily) for ventricular ectopy.

The baseline ECG in sinus rhythm showed right bundle branch block (QRS duration 126 ms) with intermittent PR prolongation (250 ms).

## Management

The patient was referred for an electrophysiology study and ablation before percutaneous pulmonary valve implantation.

**Figure 1:**
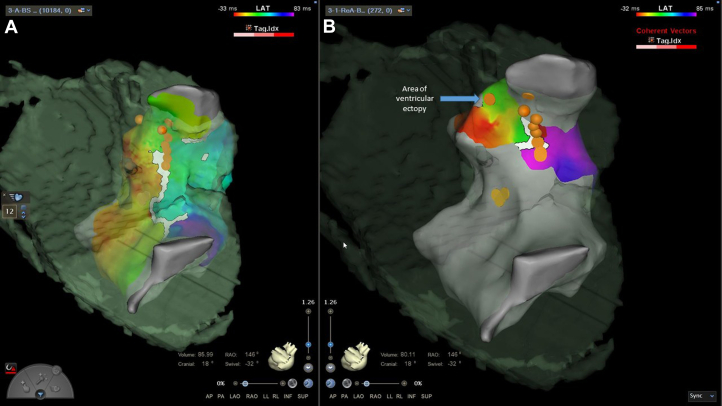
Activation Map of the Right Ventricle Posterior projections showing early to late activation mapping, demonstrating isthmus 3 below the pulmonary annulus and the region of ablation. (A) Activation map before ablation with overlying ablation points. (B) Activation map postablation with overlying ablation points.

**Figure 2:**
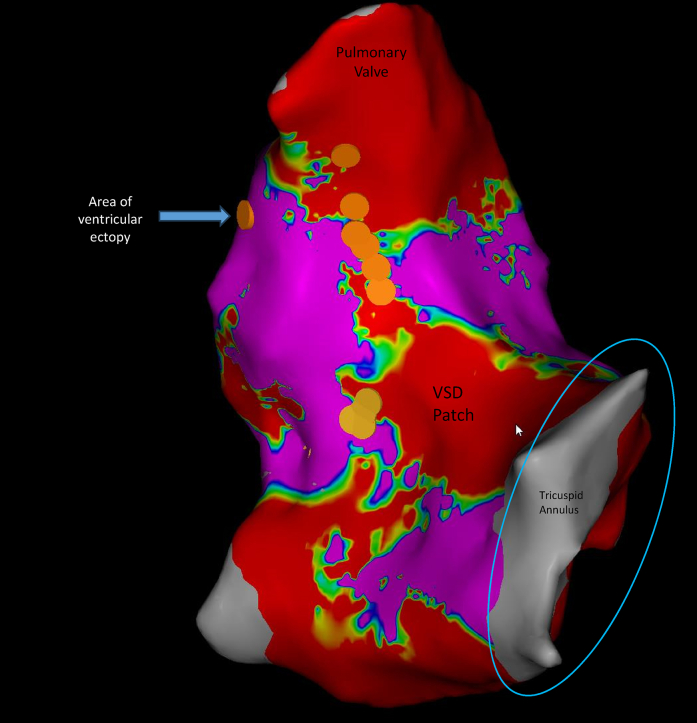
Voltage Map of the Right Ventricle Showing the Regions of Ablation Voltage mapping showing the region of slow conduction between the ventricular septal defect (VSD) patch and the pulmonary annulus as well as the area where ablation was delivered.

**Figure 3:**
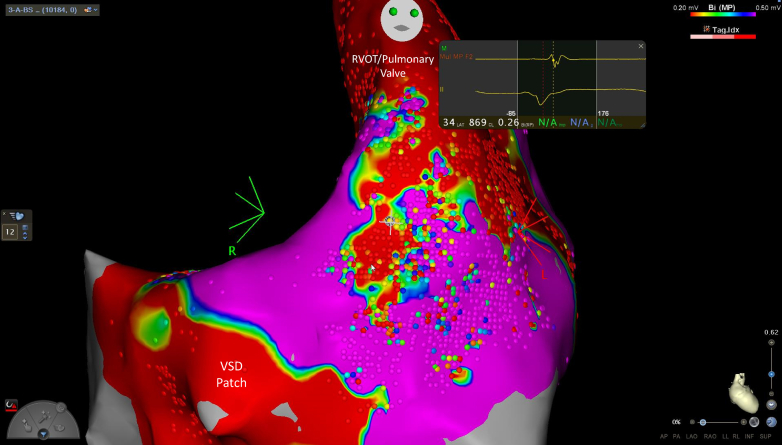
Voltage Map of the Right Ventricle Showing the Region of Slow Conduction Over Isthmus 3 Voltage mapping showing the region of slow conduction between the ventricular septal defect (VSD) patch and the pulmonary annulus. RVOT = right ventricular outflow tract.

At the outset of the procedure, the patient had frequent ectopy with couplets and triplets, and no pharmacological induction was necessary during the procedure. Activation mapping and voltage mapping were performed in the right ventricle, demonstrating a large scare corresponding to the transannular patch in the anterior RVOT and a slow conduction zone with late potentials between the scar and the region of the VSD patch/tricuspid annulus (Figures 1, 2, and 3). Five linear focal applications of PFA were delivered over this region (22 A for 4 seconds, energy delivery duration 2.4 ms, target contact force >5 g, constant irrigation flow 4 mL/min, biphasic monopolar energy application; CardioFocus Centauri focal PFA system). The standard protocol was used as previously described.^6^ Preprocedural computer tomography demonstrated the left anterior descending artery posterior and inferior to the pulmonary outflow tract. The ECG was closely inspected for ST-segment changes during the procedure, but intravenous nitrates were not needed. Block over the anatomical isthmus was demonstrated by reperforming activation mapping and pacing on both sides of the ablation line (Figure 1B). A further single application of PFA was delivered to a separate region of ventricular ectopy, with a change in the morphology of the ectopic beats. A right ventricular stimulation protocol was performed without induction of ventricular arrhythmia (3 extrastimuli from the right ventricular apex and outflow tract, with a cycle length of 510 ms for S2 and 440 ms for S3). There were no complications after the procedure.

## Follow-Up

There were no postprocedure complications. Two months after the procedure, a 3-day follow-up Holter ECG showed a 0.45% premature ventricular complex burden of different morphologies. The patient subsequently underwent successful percutaneous PVR.

## Discussion

To our knowledge, this is the first reported use of PFA for the treatment of ventricular ectopy/VT in a patient with repaired TOF. This case illustrates the convergence of 2 evolving paradigms in congenital electrophysiology: 1) the growing advocacy for pre-emptive VT substrate ablation before PVR; and 2) the emergence of PFA as a tissue-selective, nonthermal energy modality for cardiac ablation.

### Ablation prior to PVR: Timing matters

Ventricular arrhythmias remain a significant cause of late morbidity and mortality in patients with repaired TOF, largely because of macro-re-entrant circuits that develop in anatomical and surgical isthmuses—most commonly isthmus 3 (located between the pulmonary valve annulus and the VSD patch).^7^ As patients survive to older age, degeneration of the pulmonic valve requiring intervention becomes more frequent.^8^ Importantly, PVR can render the area of isthmus 3 inaccessible from an endocardial approach, potentially precluding future catheter ablation.^6^ Preoperative electrophysiology study and ablation have been advocated by Bouyer et al^3^ for risk stratification and targeted ablation, with potential reduction in postoperative arrhythmia burden.

### Pulsed field ablation: A new frontier

PFA represents a novel nonthermal ablation modality that uses high-voltage pulsed electric fields to induce irreversible electroporation, resulting in targeted myocardial cell death while minimizing injury to surrounding structures. This stands in contrast to traditional thermal ablation methods, such as RF ablation or cryoablation, which carry a risk of collateral damage to critical adjacent tissues, including coronary arteries, nerves, and cardiac valves.^4^

Preclinical and early clinical studies suggest that PFA offers several advantages: high tissue selectivity, less dependence on catheter contact force, and the ability to create durable lesions in challenging substrates, such as trabeculated or surgically altered myocardium.^4^ Although the majority of PFA research to date has focused on pulmonary vein isolation for atrial fibrillation, recent studies have begun exploring its use for VT, with early data reported by Della Rocca et al^9^ and Lozano-Granero et al.^5^

Recent developments in point-by-point PFA using energy levels of 22–25 A have further expanded the potential applications of this technology. These protocols allow precise focal lesion delivery without significant temperature elevation, enhancing procedural safety.^9^ This approach may be particularly well-suited for ablating narrow critical isthmuses, such as those encountered in patients with TOF, where precision and avoidance of collateral damage are paramount. In our experience, PFA was a quicker, more stable, and easier application than traditional RF ablation, in which longer applications in unusual anatomical positions can be challenging.

Despite its promise, PFA remains an emerging technology. Key aspects, including lesion depth, recurrence rates, and long-term safety in ventricular tissue, require further evaluation, especially in younger patients with congenital heart defects.

The present case adds to the limited but growing body of evidence supporting the feasibility of PFA for VT substrate modification in complex congenital heart disease. Specifically, it demonstrates successful PFA delivery in a patient with TOF, highlighting the potential role of this technology in expanding treatment options for arrhythmia management in this unique population.

## Funding Support and Author Disclosures

Prof Sabine Ernst is a consultant for Biosense and Stereotaxis. All other authors have reported that they have no relationships relevant to the contents of this paper to disclose.

**Take-Home Messages**

• This case demonstrates the feasibility of using focal pulsed field ablation for the treatment of ventricular tachycardia in patients with tetralogy of Fallot.
• It highlights the role of pre-emptive substrate modification before pulmonary valve replacement in patients with tetralogy of Fallot.

## Appendix

Supplemental Figure 1 Baseline ECG Before Exercise With Ventricular Ectopy

Supplemental Figure 2. ECG During Exercise With Nonsustained Ventricular Tachycardia and Ventricular Ectopy

## Footnotes

Appendix For supplemental figures, please see the online version of this paper.

**Visual Summary:** Timeline of the Case

Timeline	Events
Case history	A 45-year-old man with repaired tetralogy of Fallot presented with severe pulmonary regurgitation and enlarged right ventricular volumes and was referred for pulmonary valve replacement. He was clinically well and noted to have ventricular ectopy that was exacerbated by exercise.
EPS	An EPS was performed pre-emptively before pulmonary valve implantation, which identified a slow conduction zone at isthmus 3 (located between the RVOT and the VSD patch—the region typically covered by pulmonary valve implantation). This region was ablated using focal PFA without complications.
Pulmonary valve replacement	Two months after the procedure, a 3-day follow-up Holter ECG showed a minimal premature ventricular complex burden (0.45%). The patient subsequently underwent successful percutaneous pulmonary valve replacement.

ECG = electrocardiogram; EPS = electrophysiology study; PFA = pulsed field ablation; RVOT = right ventricular outflow tract; VSD = ventricular septal defect.
