# Supplementary material for: Pulsed Field Ablation for Ventricular Arrhythmia in Repaired Tetralogy of Fallot
Source: JACC Case Rep. 2025 Dec 11;31(5):106335. doi: 10.1016/j.jaccas.2025.106335 (PMC12881302; doi:10.1016/j.jaccas.2025.106335)

Supplementary Figure 1: Baseline ECG prior to exercise with ventricular ectopy


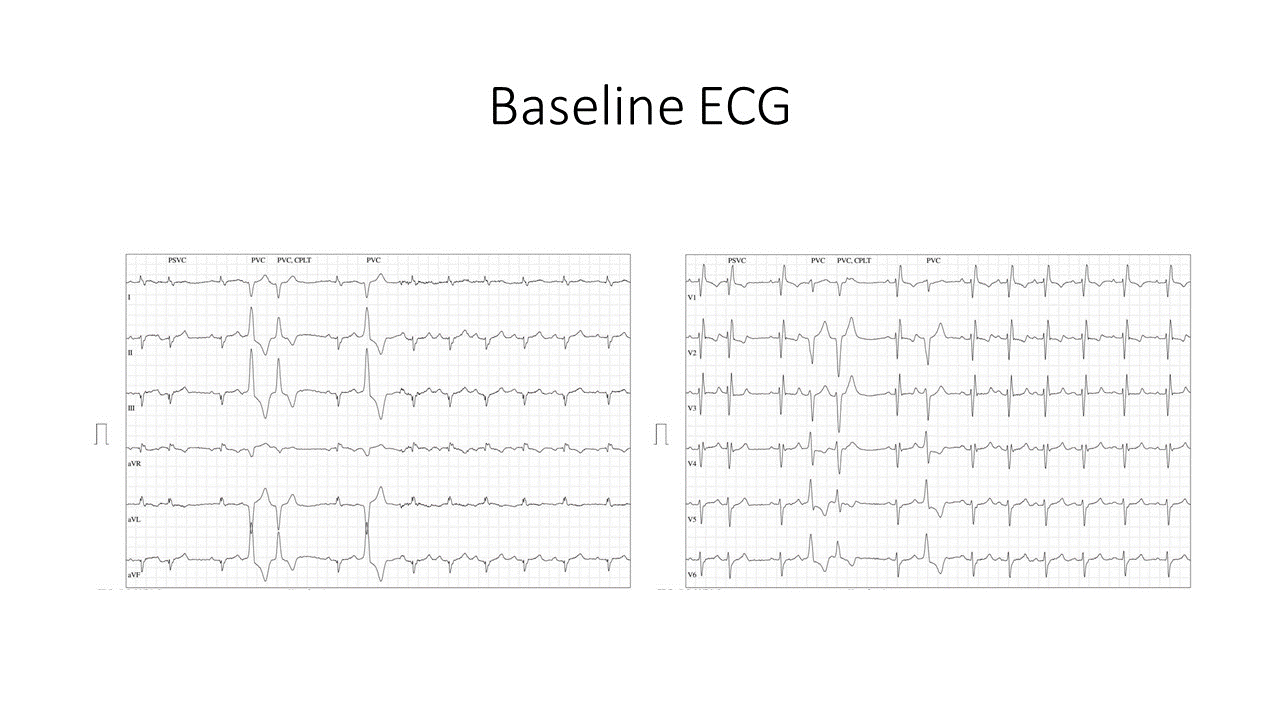


Supplementary Figure 2: ECG during exercise with non-sustained ventricular tachycardia and ventricular ectopy


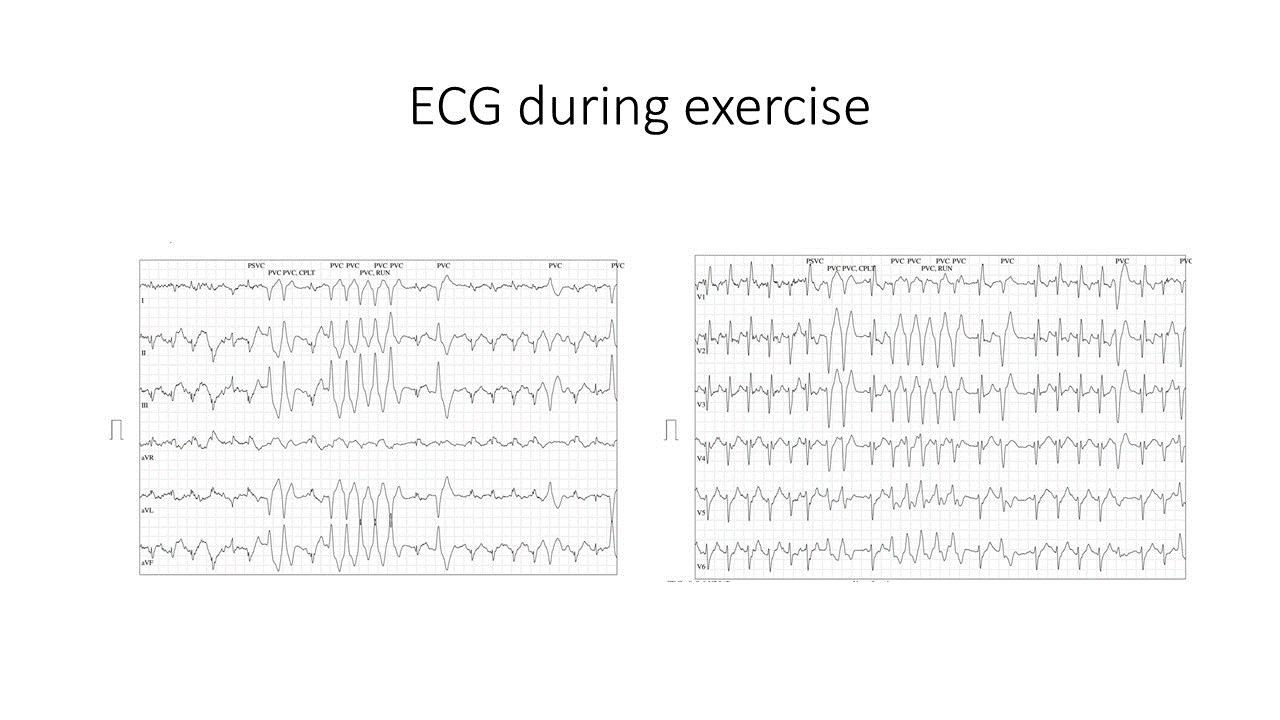

Supplement: Supplemental Figure 1 — Baseline ECG Before Exercise With Ventricular Ectopy [file mmc1.docx]
